# Transmissible Gastroenteritis Virus Infection Up-Regulates FcRn Expression via Nucleocapsid Protein and Secretion of TGF-β in Porcine Intestinal Epithelial Cells

**DOI:** 10.3389/fmicb.2019.03085

**Published:** 2020-01-21

**Authors:** Shaoju Qian, Zitong Gao, Rui Cao, Kang Yang, Yijie Cui, Shaowen Li, Xianrong Meng, Qigai He, Zili Li

**Affiliations:** ^1^State Key Laboratory of Agricultural Microbiology, College of Veterinary Medicine, Huazhong Agricultural University, Wuhan, China; ^2^Key Laboratory of Preventive Veterinary Medicine in Hubei Province, The Cooperative Innovation Center for Sustainable Pig Production, Wuhan, China; ^3^Key Laboratory of Development of Veterinary Diagnostic Products, Ministry of Agriculture of the People’s Republic of China, Wuhan, China

**Keywords:** transmissible gastroenteritis virus, nucleocapsid protein, TGF-β, neonatal Fc receptor, toll-like receptor, NF-κB

## Abstract

Transmissible gastroenteritis virus (TGEV) is a porcine intestinal coronavirus that causes fatal severe watery diarrhea in piglets. The neonatal Fc receptor (FcRn) is the only IgG transport receptor, its expression on mucosal surfaces is triggered upon viral stimulation, which significantly enhances mucosal immunity. We utilized TGEV as a model pathogen to explore the role of FcRn in resisting viral invasion in overall intestinal mucosal immunity. TGEV induced FcRn expression by activating NF-κB signaling in porcine small intestinal epithelial (IPEC-J2) cells, however, the underlying mechanisms are unclear. First, using small interfering RNAs, we found that TGEV up-regulated FcRn expression via TLR3, TLR9 and RIG-I. Moreover, TGEV induced IL-1β, IL-6, IL-8, TGF-β, and TNF-α production. TGF-β-stimulated IPEC-J2 cells highly up-regulated FcRn expression, while treatment with a JNK-specific inhibitor down-regulated the expression. TGEV nucleocapsid (N) protein also enhanced FcRn promoter activity via the NF-κB signaling pathway and its central region (aa 128–252) was essential for FcRn activation. Additionally, N protein-mediated FcRn up-regulation promotes IgG transcytosis. Thus, TGEV N protein and TGF-β up-regulated FcRn expression, further clarifying the molecular mechanism of up-regulation of FcRn expression by TGEV.

## Introduction

Transmissible gastroenteritis virus (TGEV) infection mainly causes severe watery diarrhea, vomiting, dehydration, and mortality in suckling piglets less than 2 weeks old, as well as colossal economic losses in the worldwide pig industry (Zhao et al., 2014). TGEV has a 28.5-kb single-stranded, positive-sense RNA genome, including at least nine open reading frames (ORFs): ORF1a, ORF1b, Structural proteins, nucleocapsid (N) protein, membrane (M) glycoprotein, spike (S) glycoprotein, a small envelope (E) glycoprotein, and accessory proteins 3a, 3b, and 7 (Escors et al., 2003). ORF1a and ORF1b, located in the 5′ two-thirds of the viral genome, are cleaved into 16 non-structural proteins (nsp1 to nsp16) by the nsp3 and nsp5 proteases. These nsps are responsible for viral replication, viral transcription, and the antagonization of host innate immune responses.

Transmissible gastroenteritis virus replication occurs in the small intestinal epithelial cells, while viral entry and release are restricted to the apical surface of polarized epithelial cells. Neonatal Fc receptor (FcRn) is a specific receptor for the immunoglobulin IgG, which is expressed in many cells, including epithelial cells, macrophages, and dendritic cells (Ye et al., 2008). FcRn transports of IgG in the female reproductive tract plays an important role in combating infection (Li et al., 2011). In addition to IgG, FcRn binds albumin, which regulates liver damage (Pyzik et al., 2017). It has been found that FcRn can enhance HIV-I endocytosis in transmucosal epithelial cells (Gupta et al., 2013). It has also been documented that the use of fusion proteins of the Fc fragment as immunogenic antigens can improve the impact of vaccines. Intranasally inoculated fusion protein, HSV-2 gD, HIV Gag fused with the Fc region of IgG, and targeted FcRn can all induce systemic and mucosal immunity to genital infections (Lu et al., 2011; Ye et al., 2011). Most studies on FcRn have focused on the function of FcRn in humans and mice; so far, only a few relevant studies about pathogenic infection and FcRn expression regulation have been reported. In our previous study, TGEV induced FcRn expression via the NF-κB pathway in IPEC-J2 cells (Guo et al., 2016b), but the regulatory mechanisms underlying this remain unclear; thus, we need to further explore the involvement of pattern recognition receptors (PRRs), inflammatory factors, and virus-coding proteins in the regulation of FcRn.

In the innate immune system, PRRs recognize pathogen-associated molecular patterns (PAMPs) as the first step in the host’s resistance to infection. After recognition of PAMPs, these receptors interact with their corresponding binding molecules to trigger downstream signaling events that activate NF-κB and IFN regulatory factors that induce several types of antiviral cytokines (Thompson and Locarnini, 2007). TGEV infection activates transcriptional activator 1 (JAK-STAT1) signaling pathways according to a quantitative proteomics study in PK-15 cells (An et al., 2014). TGEV infection has been reported to activate the mitogen-activated protein kinase (MAPK) pathway and destroy epithelial barrier integrity in IPEC-J2 cells (Zhao et al., 2014). TGEV N, nsp2 and nsp14 proteins all induce NF-κB activation (Zhou et al., 2017; Wang et al., 2018; Zhang et al., 2018), but the regulatory mechanism of FcRn is still unknown. Most studies on TGEV have focused on the induction and activation of NF-κB and IFN, but host cell biology may affect cell function. However, the detailed mucosal immunity mechanism during TGEV pathogenesis remains unknown.

Viral invasion always triggers an inflammatory response, which is the key mediator of host response to microbial pathogens (McDonnell et al., 2016). TGEV infection has been shown to induce the production of IFNs and pro-inflammatory cytokines *in vitro* and *in vivo* (Cruz et al., 2013). A direct correlation between dsRNA antiviral response induction and TGEV virulence has been demonstrated (Cruz et al., 2011). Moreover, the inflammatory factors produced will also contribute to the production of a strong immune response. TNF-α and IL-1β can activate NF-κB to up-regulate the expression of human FcRn, which enhances FcRn-mediated IgG transport (Liu et al., 2007). TGEV infection was also found to induce EMT via TGF-β in IPEC-J2 cells (Xia et al., 2017). The aim of this study was to identify the TGEV-encoded proteins involved in inducing FcRn production.

## Materials and Methods

### Cells, Virus, and Antibodies

IPEC-J2 cells donated by Xiaoping Li from Huazhong Agricultural University (Hubei Province, Wuhan, China) were cultured in Dulbecco’s modified Eagle’s medium (DMEM; Hyclone, United States) containing 10% fetal bovine serum (FBS; Gibco, United States) and 1% penicillin/streptomycin at 37°C in a 5% CO_2_ atmosphere. The isolated TGEV strain WH-1 (GenBank HQ462571) was propagated in IPEC-J2 cells. In our laboratory, AffiniPure rabbit anti-cytoplasmic tails of porcine FcRn (anti-FcRn-CT) polyclonal antibodies were prepared (Guo et al., 2016a). Horseradish peroxidase (HRP)-conjugated goat anti-rabbit and goat anti-mouse IgG were purchased from Abclona (China). Monoclonal antibody (mAb) against GAPDH in mice was purchased from Abclona (China). Mouse mAbs against HA, IκBα, NF-κB, p65 and rabbit mAbs against phospho-NF-κB and p65 were obtained from Cell Signaling Technology (United States).

### Plasmid Construction and siRNA

Luciferase reporter plasmids FcRn-Luc, NF-κB-Luc, pR-TK, p65-EGFP, and p65-Tag2B were prepared in our laboratory and have been described previously. Genes encoding TGEV proteins were amplified from the genomic RNA of the TGEV strain WH-1 and then cloned into the expression vector pCAGGS-HA (Guo et al., 2016b) (Table 1). pCAGGS-N was used as a template to amplify several deletion mutants of the virus N gene. These mutants were then cloned into the pCAGGS-HA (Table 2). Small interfering RNA (siRNA) molecules targeting TLR2, TLR3, TLR4, TLR8, TLR9, RIG-I, MyD88, TRIF, and negative controls were obtained from Shanghai GenePharma (Table 3). MAPK inhibitors SB203580, SP600125, U0126 and DMSO were purchased from Sigma-Aldrich (United States).

**TABLE 1:** Sequences of primers for cloning the TGEV genomic fragments.

**Primer**	**Sequence (5′–3′)**	**Restriction enzyme site**
nsp1-F	GCGGTACCATGAGTTCCAAACAATTCAAG	*Kpn*I
nsp1-R	ATTAGATCTACCTCTGCCAGTGCGAGCAAT	*Bgl*II
nsp2-F	GGCGGTACCGCCATATATGTTGATCAATA	*Eco*RI
nsp2-R	GCCCTCGAGACCACCCATTTTATTATAC	*Xho*I
nsp3-F	GGCGGTACCGGTGACAAAACTGTCTCAT	*Kpn*I
nsp3-R	GCCCTCGAGACCACTTTTTGGAGACACA	*Xho*I
nsp4-F	CTCGGTACCTCAGGCTTTTTCGATGTAAT	*Kpn*I
nsp4-R	CGACTCGAGCTGAAGTGTAGAATTAACAC	*Xho*I
nsp5-F	GCGAATTCTCAGGTTTGCGGAAAATGGC	*Xho*I
nsp5-R	CGGGGTACCCTGAAGATTTACACCATAC	*Bgl*II
nsp6-F	GGCGGTACCGCTGGTAAAGTAAAATCTTT	*Kpn*I
nsp6-R	GGCCTCGAGCTGTACAGTTGAAATTTTG	*Xho*I
nsp7-F	GCCGGTACCTCAAAACTTACAGAGATGAA	*Kpn*I
nsp7-R	TAGAGATCTCTGGAGTATGGTGGTGTTCT	*Bgl*II
nsp8-F	TCGAATTCAGTGTGGCTTCAGCTTATGC	*Eco*RI
nsp8-R	TAACTCGAGCTGAAGCTTTGTGGTTCTCT	*Xho*I
nsp9-F	CCGGTACCAACAATGAAATTATGCCAGG	*Kpn*I
nsp9-R	TACTCGAGTTGCAGACGAACTGTTGCA	*Xho*I
nsp10-F	CGGAATTCGCTGGTAAACCCACTGAA	*Eco*RI
nsp10-R	TGCTCGAGATAGAAGTACGATCGCACATG	*Xho*I
nsp11-F	CGGGGTACCATGAGTTTTACTGTTGATCAA	*Kpn*I
nsp11-R	AAGCTCGAGTGTTGCAGCAATTGACTT	*Xho*I
nsp12-F	ATTGGTACCTAGAACCCTGCAATGGTACT	*Kpn*I
nsp12-R	CAACTCGAGTTGCAAGACAGTGGACTTTT	*Xho*I
nsp13-F	TAAGAATTCGCTGCAGGCATGTGTGTAGT	*Eco*RI
nsp13-R	CGGCTCGAGTTGTAAACCAATCTTTGAA	*Xho*I
nsp14-F	GCGAATTCGCAAAACCTGAAACTTGTGG	*Eco*RI
nsp14-R	TGGCTCGAGCTGAAGTGCTTTGCTATTAA	*Xho*I
nsp15-F	GCCGGTACCAGTCTAGAAAATGTGGCTTT	*Kpn*I
nsp15-R	AGCTCGAGTTGGAGTTGTGGATAGAAGG	*Xho*I
nsp16-F	TAAGAATTCTCTGCTGAATGGAATCCCGG	*Eco*RI
nsp16-R	ATACTCGAGTGGTGTGTTAACGAAGTGGT	*Xho*I
S1-F	CCG*GAATTCGCCATG*AAAAAACTATTTGTGG	*Eco*RI
S1-R	CCAACTAT*GGTACC*ATCAATAACAGCTGC	*Kpn*I
S2-F	GCAGCTGTTATTGAT*GGTACC*ATAGTTGG	*Kpn*I
S2-R	GCG*GTCGAC*AATTTAATGGACGTGCACT	*Sal*I
M-F	GCGGGTACCATGAAGATTTTGTTAATA	*Kpn*I
M-R	GCGCTCGAGTAGTTATACCATATGTAA	*Xho*I
N-F	CATGAATTCATGGCCAACCAGGGACAAC	*Eco*RI
N-R	CACCTCGAGTTAGTTCGTTACCTCATC	*Xho*I
E-F	CTCGAATTCATGACGTTTCCTAGGGCATTG	*Eco*RI
E-R	ACTAGATCTTCAAGCAAGGAGTGCTCCATC	*Bgl*II
3b -F	GCCCTCGAGATGATTGGTGGACTTTTTCTT	*Eco*RI
3b -R	CGGGAATTCCTAGGAAACGTCATAGGTATG	*Xho*I

**TABLE 2:** Sequences of primers used for cloning the TGEV N-protein deletion mutants.

**Primer**	**Sequence (5′–3′)**	**Restriction enzyme site**
ΔN1-F	CATGAATTCATGGCCAACCAGGGACAAC	*Eco*RI
ΔN1-R	CACCTCGAGAAGCGTGGTTGGTTTGTTCAT	*Xho*I
ΔN2-F	CATGAATTCATGGGTAGTCGTGGTGCTAAT	*Eco*RI
ΔN2-R	CACCTCGAGAGCTCCATAAAATCTTGTCAC	*Xho*I
ΔN3-F	CATGAATTCATGAGAAGCAGTTCAGCCA	*Eco*RI
ΔN3-R	CACCTCGAGTTAGTTCGTTACCTCATC	*Xho*I
ΔN4-F	CGCGAATTCATGAAAGCTTTGAAATTCGAT	*Eco*RI
ΔN4-R	CGCCTCGAGACGTTCTTTAGATTTAGAACG	*Xho*I

**TABLE 3:** Sequences of siRNA used in the study.

**Gene name**	**siRNA sequence (sense)**	**siRNA sequence (anti-sense)**
siTLR2	GCC CUU CCU ACA CAC UUU ATT	UAA AGU GUG UAG GAA GGG CTT
siTLR3	GCUUAAGUGUGAUUGGU AATT-	UUACCAAUCACACUUAA GCTT
siTLR4	GCAUGGAGCUGAAUUUC UATT	UAGAAAUUCAGCUCCAU GCTT
siTLR8	GCUGGAAGACAACCAGU UATT	UAACUGGUUGUCUUCCA GCTT
siTLR9	GCCUCUCCUUACUCUCC AATT	UUGGAGAGUAAGGAGAG GCTT
siRIG-I	GGUACAAAGUUGCAGG CAUTT	AUGCCUGCAACUUUGUA CCTT
siMyD88	CAG CUG AGA AGC CUU UAC ATT	UGU AAA GGC UUC UCA GCU GTT
siTRIF	GGA GUU AUC GGA ACA GAA ATT	UUU CUG UUC CGA UAA CUC CTT
Negative control	UUC UCC GAA CGU GUC ACG UTT	ACG UGA CAC GUU CGG AGA ATT

### RNA Extraction and Real-Time RT-qPCR

Total RNA was prepared using TRIzol^@^ reagent (Invitrogen). RT-qPCR was performed using SYBR Green PCR Master Mix (Takara, Osaka, Japan) in the Bio-Rad Sequence Detection System (Bio-Rad). Each transcript from each sample was determined three times. The delta threshold cycle (2^–ΔΔCT^) method was used to calculate the fold-change compared to the normal gene expression. The specific pig gene primers (Table 4) were designed using Primer Express software (version 3.0; Applied Biological Systems, Carlsbad, CA, United States) as previously described with some modifications (Temeeyasen et al., 2018).

**TABLE 4:** Sequences of the primers used for RT-qPCR.

**Gene name**	**sequence (sense)**	**sequence (anti-sense)**
TLR2	TCATCTCCCAAATCTGCGAAT	GGCTGATGTTCTGAATTG ACCTC
TLR3	GCGGTCCTGTTCAGTTTCT	AAGGCATCTGCTGGGATTT
TLR4	CCTGCCTGTGCTGAGTTTCA	AAGGTGAGAACTGACGCAC TAATG
TLR8	CTTTGATGATGACGCTGC TTTC	GGTGTGTCACTCCTGCT ATTC
TLR9	CCTCACACATCTCTCACT CAAG	GGTGACAATGTGGTTGT AGGA
RIG-I	GAGCCCTTGTGGATGCTTTA	GGGTCATCCCTATGTTCTG ATTC
MyD88	GGCAGCTGGAACAGACCAA	GGTGCCAGGCAGGACATC
TRIF	ACTCGGCCTTCACCATCCT	GGCTGCTCATCAGAGACT GGTT
FcRn	GGCGACGAGCACCACT ACTG	AGCCGACCATGATTCCA ACC
TGF-β	ACGTGGAGCTATACCAGAAAT ACAG	ACAACTCCGGTGACATCA AAGG
IL-1β	GGCCGCCAAGATATAACTGA	GGACCTCTGGGTATGGC TTTC
IL-6	AACCTGAACCTTCCAAAA ATGG	ACCGGTGGTGATTCTCA TCA
IL-8	AGTTTTCCTGCTTTCTGCA GCT	TGGCATCGAAGTTCTGC ACT
TNF-α	TCCACCAACGTTTTCCTCACT	AGGGCTCTTGATGGCA GAGA
GAPDH	CCCCAACGTGTCGGTTGT	CCTGCTTCACCACCTTC TTGA

### Western Blot and Porcine Cytokine Array

The samples were harvested with lysis buffer (4% sodium dodecyl sulfate, 3% dithiothreitol, 65 mM Tris–HCl [pH 6.8], and 30% glycerol). Proteins were separated via SDS-PAGE and then electrotransferred onto a polyvinylidene difluoride membrane (Bio-Rad, United States). Western blot analysis was performed using the indicated antibodies following the procedure as described previously (Guo et al., 2016b).

IPEC-J2 cells grown in a six-well plate were infected with TGEV, the supernatant of IPEC-J2 cells was harvested, and IL-1β, IL-6, IL-8, TGF-β, and TNF-α protein levels were measured with a Quantibody Porcine Cytokine Array 1 (Ray Biotech, Norcross, GA, United States) according to the manufacturer’s instructions.

### Transfection and Dual-Luciferase Assay

IPEC-J2 cells at 80% confluence were co-transfected with 0.2 μg FcRn-Luc and 0.1 μg pRL-TK using Lipofectamine 2000 (Invitrogen) reagent before treatment with TGEV, specific siRNA molecules, or recombinant plasmids. Cell lysates were collected at the indicated time points over 36 h and measured using a dual-luciferase assay kit (Promega).

### Eukaryotic Expression of TGEV N Protein and IgG Transcytosis

The recombinant TGEV N protein were prepared using a baculovirus/insect cell expression system as previously described (Chen et al., 2017). IPEC-J2 cells were added (0, 10, 20, 50, 100 or 200 ng/mL) recombinant TGEV N protein in medium and its FcRn expression detected by RT-qPCR and Western blot.

IgG transport was performed as previously described methods (Guo et al., 2016a). Transepithelial electrical resistance (TEER) was measured with planar electrodes (World Precision Instruments). IPEC-J2 cells were grown on 0.4-μm pore size transwell filter inserts (Corning Costar, United States) to form a monolayer exhibiting TEER > 1000 Ω/cm^2^ about 6–7 days. Monolayers were stimulated with recombinant TGEV N protein (100 ng/mL) for 12 h. Thereafter, Biotin-IgG (200 μg/mL) were applied to the basolateral or apical DMEM medium and incubated for 3 h at 37°C. Meanwhile, the opposite chamber was incubated in DMEM medium. Three hours later, samples were collected in which apically and basolaterally directed IgG transports were conducted. Subsequently, the bidirectional transport (apical-to-basolateral or basolateral-to-apical) of IgG were measured by Western blot and avidin blot analysis.

### Statistical Analysis

All experiments were repeated a minimum of three times. Differences among groups were determined by one-way ANOVA using GraphPad Prism (version 5.0; GraphPad Software). The significance levels for all analyses were set as *p* < 0.05 (^∗^) and *p* < 0.01 (^∗∗^).

## Results

### TGEV-Induced FcRn Activation Is Closely Related to Viral Replication

**FIGURE 1:**
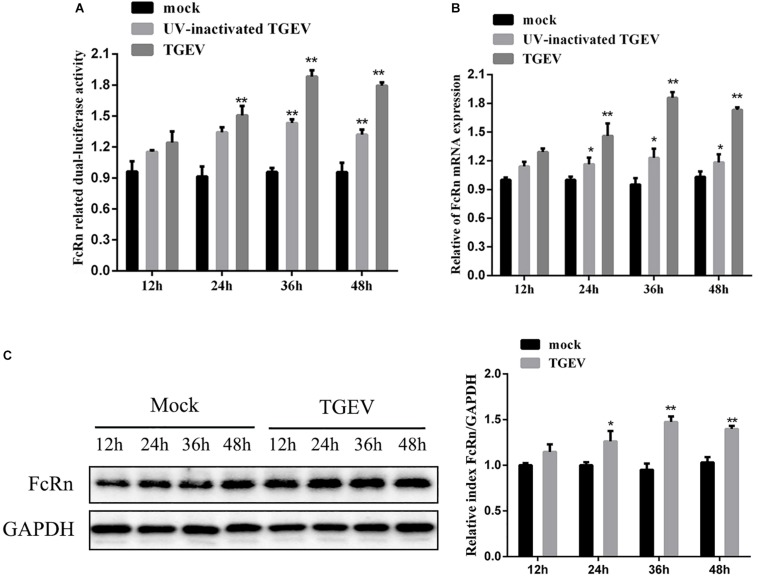
TGEV-induced activation of FcRn depends on viral replication. **(A)** IPEC-J2 were co-transfected with pRL-TK and FcRn-Luc, followed by alive TGEV or UV-inactivated TGEV (MOI 1). Cells were harvested at 12, 24, 36 or 48 hpi, and the lysates were analyzed by dual-luciferase assay. **(B)** IPEC-J2 cells were infected with alive TGEV or UV-inactivated TGEV at a MOI of 1. Cells were harvested at 12, 24, 36 or 48 hpi, and the lysates were analyzed by RT-qPCR. **(C)** IPEC-J2 were infected with alive TGEV at a MOI of 1. Cells were harvested at 12, 24, 36 or 48 hpi, and the lysates were analyzed by Western blot. ^∗^*p* < 0.05, ^∗∗^*p* < 0.01.

To examine if TGEV infection induces FcRn promoter activation, IPEC-J2 cells were co-transfected with FcRn-luc or pRL-TK. Twenty-four hours later, the cells were incubated with live or UV-inactivated TGEV for 12, 24, 36, and 48 h post-infection (hpi). Enhanced FcRn luciferase activities tended to increase during the progression of TGEV infection from 24 to 48 hpi, and this increase was significantly different compared with mock-infected IPEC-J2 cells (Figure 1A). Therefore, the up-regulation of FcRn is closely related to the replication of TGEV.

In order to determine whether TGEV induces FcRn expression in IPEC-J2 cells, live or UV-inactivated TGEV at a multiplicity of infection (MOI) of 1 was used to inoculate IPEC-J2 cells, which were then harvested for FcRn analysis at 12, 24, 36, and 48 hpi by RT-qPCR and Western blot (Figures 1B,C). TGEV induced FcRn mRNA expression about two fold higher at 24, 36, and 48 hpi, and the elevation in protein levels correlated with the increased mRNA. However, UV-inactivated viral treatment resulted in only a 1.3-fold up-regulation of FcRn expression compared with that in simulated infected cells, indicating that the replicating virus is more effective than the UV-inactivated virus.

### Role of TLR and NF-κB in TGEV-Mediated Activation of FcRn

**FIGURE 2:**
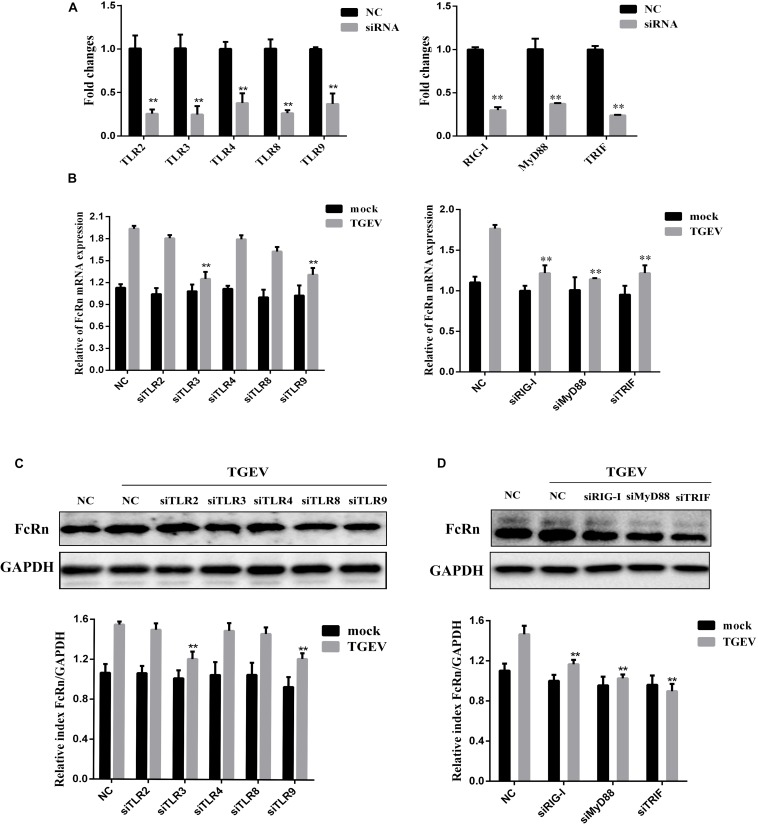
Involvement of TLR signaling cascades in FcRn activation by TGEV. **(A)** IPEC-J2 cells were transfected with 50 nM specific siRNAs targeting TLR2/3/4/8/9, TRIF, MyD88, RIG-I or NC siRNA, respectively for 24 h, then cells were collected for analysis of mRNA levels using RT-qPCR assays. **(B)** IPEC-J2 cells were transfected with the siRNAs TLR2/3/4/8/9, TRIF, MyD88, RIG-I or NC siRNA, respectively for 24 h, then with/without TGEV infection (MOI = 1). At 24 hpi, total RNA was extracted and FcRn or GAPDH mRNA expression was analyzed by RT-qPCR. **(C,D)** IPEC-J2 cells were transfected with the siRNAs TLR2/3/4/8/9, TRIF, MyD88, RIG-I or NC siRNA, respectively for 24 h, then with/without TGEV infection (MOI 1). IPEC-J2 cells were harvested at 36 hpi and measured by Western blot. The right panel represents the quantification of the bands by densitometry, corrected by the amount of GAPDH. ^∗∗^*p* < 0.01.

The small intestine of pigs expressed different TLRs, such as TLR1, TLR2, TLR3, TLR4, TLR6, TLR8, TLR9, and TLR10 (Arce et al., 2010). It has been reported that viral nucleic acids are recognized by TLR3, TLR8, and TLR9, whereas viral proteins are recognized by TLR2 and TLR4 (Thompson and Locarnini, 2007). To determine whether TLRs are involved in mediating TGEV-induced FcRn activation, specific siRNAs were synthesized to target binding molecules in the TLR2, TLR3, TLR4, TLR8, TLR9, TRIF, MyD88, and RIG-I pathways. Transient transfection and RT-qPCR assays proved the low knockout efficiency of each siRNA, and the silencing efficiencies of the siRNAs to these receptors were over 50% (Figure 2A). The cells were transfected with each siRNA and then infected with TGEV; then, the changes in FcRn expression were detected by RT-qPCR and Western blot. The results showed that, compared with the cells transfected with NC siRNA, siRNA knockdown of TLR3, TLR9, RIG-I, TRIF, and MyD88 effectively reduced the TGEV-induced up-regulation of FcRn, while FcRn mRNA expression was not inhibited in cells transfected with siRNA targeting TLR2, TLR4, or TLR8 (Figure 2B). Next, IPEC-J2 cells were transfected with TLR-specific siRNA or NC siRNA. After 12 h, the cells were infected with 1 MOI TGEV for an additional 36 h. Compared with NC siRNA, TLR3, TLR9, RIG-I, TRIF, and MyD88-specific siRNAs inhibited TGEV-induced FcRn expression, while no significant change was detected in s TLR2, TLR4 and TLR8-specific siRNAs (Figures 2C,D). These data suggest that TGEV infection activates FcRn via TLR3, TLR9, TRIF, MyD88, and RIG-I.

### TGF-β Stimulated by TGEV Promotes the Expression of FcRn

**FIGURE 3:**
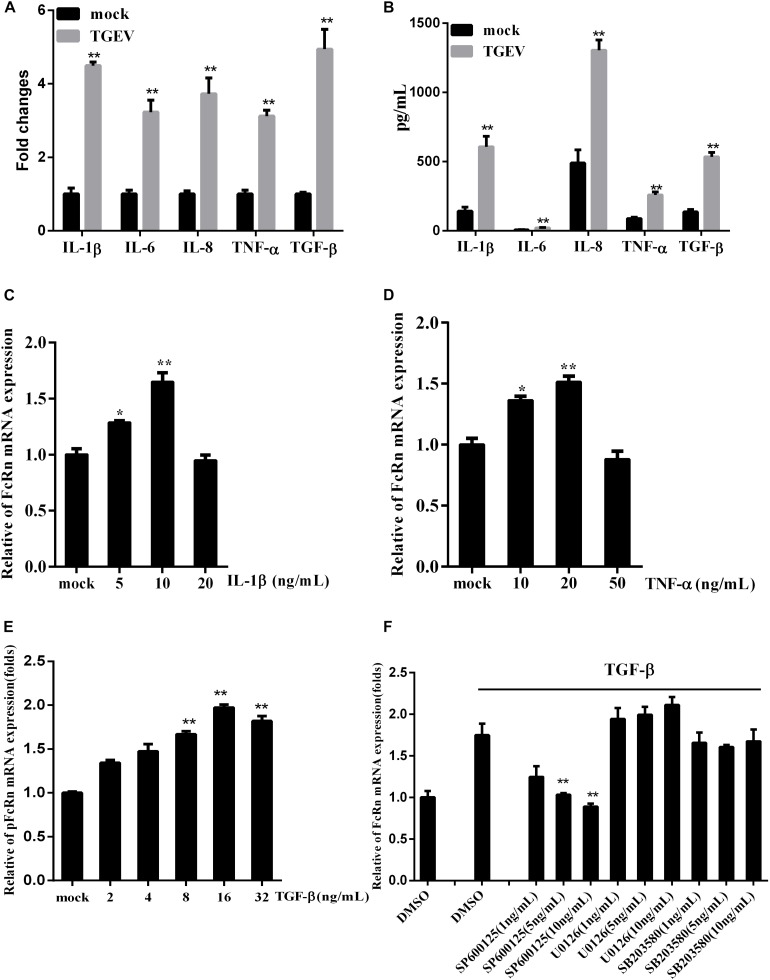
Regulation of FcRn mRNA on IPEC-J2 cells stimulated by TGF-β. **(A,B)** The relative mRNA and protein levels of IL-1β, IL-6, IL-8, TNF-α, and TGF-β in IPEC-J2 infected with TGEV for 36 hpi by RT-PCR and porcine cytokine array. **(C–E)** IPEC-J2 cells were stimulated with TNF-α, IL-1β, TGF-β for 2 h, and the levels of FcRn mRNA were detected using RT-qPCR. **(F)** IPEC-J2 cells were pretreated with SB202190 (1, 5, and 10 ng/mL), SP600125 (1, 5, and 10 ng/mL), or U0126 (1, 5, and 10 ng/mL) or DMSO (control) for 2 h, followed by with/without TGF-β for 2 h and the levels of FcRn mRNA were detected using RT-qPCR. ^∗^*p* < 0.05, ^∗∗^*p* < 0.01.

To detect the effect of TGEV infection on inflammatory cytokines, RT-qPCR and a porcine cytokine array were used to detect the expression levels of IL-1β, IL-6, IL-8, TGF-β, and TNF-α in IPEC-J2 cells infected with TGEV at 36 h. TGEV significantly increased the expression levels of five cytokines, especially TGF-β (Figures 3A,B).

We treated IPEC-J2 cells with TNF-α and IL-1β in different concentrations. TNF-α and IL-1β induced FcRn expression in a dose-dependent manner, increasing mRNA levels 1.5–1.7-fold after 2 h (Figures 3C,D). Interestingly, we found that TGF-β can also up-regulate FcRn, increasing the mRNA level 1.9-fold after 2 h (Figure 3E). Meanwhile, IPEC-J2 cells were treated with SB202190 (p38 inhibitor), SP600125 (JNK1/2 inhibitor) and U0126 (ERK1/2 inhibitor), followed by TGF-β incubation. Treatment with SP600125 sharply reduced FcRn expression, indicating that TGF-β up-regulated FcRn expressions via the c-Jun amino-terminal kinase (JNK)-MAPK pathway in IPEC-J2 cells (Figure 3F).

### TGEV N Protein Enhances FcRn Expression via NF-κB Signaling Pathways

**FIGURE 4:**
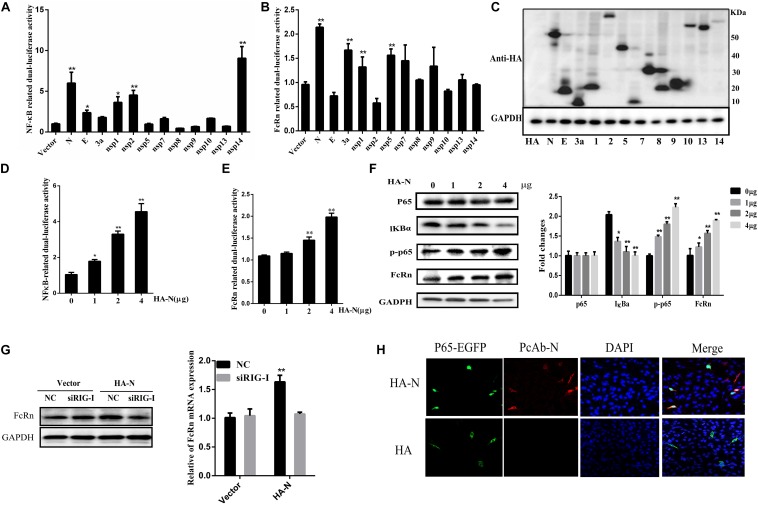
Transmissible gastroenteritis virus N induces FcRn and NF-κB activation. **(A,B)** IPEC-J2 cells were co-transfected with pRL-TK and FcRn-Luc or NF-κB-Luc together with each expression plasmid of TGEV proteins. **(C)** Lysates of transfected cells were subjected to Western blot with anti-HA antibody to detect viral protein expression. **(D,E)** IPEC-J2 were co-transfected with pRL-TK, FcRn-Luc or NF-κB-Luc, and TGEV N expression plasmids (1, 2 or 4 μg) for 36 h. The cell lysates were analyzed by dual-luciferase assay. **(F)** FcRn, IκBα, p65 and phosphorylated p65 (p-p65), and GAPDH was assayed by Western blot analysis. **(G)** IPEC-J2 cells were transfected with siRIG or NC siRNA for 24 h and subsequently retransfected with TGEV N-expression plasmids (4 μg) for an additional 24 h. FcRn expression were detected by Western blot analysis. The right panel represents the quantification of the bands by densitometry, corrected by the amount of GAPDH. **(H)** IPEC-J2 cells were co-transfected with the p65-EGFP fusion protein vector and an empty vector (HA) or the TGEV N expression vector (HA-N) for 24 h. NF-κB p65 (green), TGEV N (red), and the nuclei (blue) were visualized by confocal microscopy. ^∗^*p* < 0.05, ^∗∗^*p* < 0.01.

Because NF-κB is a necessary transcription factor for FcRn production, we examined the effects of all TGEV-encoded proteins on NF-κB promoter activity. TGEV encodes 16 non-structural proteins (nsp1–16), four structural proteins (S, E, M, and N), and three helper proteins (ORF7, ORF3a, and ORF3b), which were constructed recombinant plasmids by using the pCAGGS-HA vector. However, efficient expression occurred only for the plasmids encoding N, E, 3a, nsp1, nsp2, nsp5, nsp7, nsp8, nsp9, nsp10, nsp13, and nsp14 (Figure 4C). IPEC-J2 cells were co-transfected with NF-κB-Luc or FcRn, pRL-TK and different TGEV protein expression vectors. We found that TGEV N, nsp2 and nsp14 can all activate NF-κB (Figure 4A), and TGEV N, 3a, nsp1 and nsp5 can up-regulate FcRn by about 1.5–2-fold and N was the most significant inducer produced by FcRn (Figure 4B).

To confirm the involvement of N protein in NF-κB or FcRn promoter activation, IPEC-J2 cells were co-transfected with pRL-TK, NF-κB-Luc or FcRn-Luc and N expression vectors were added at different concentrations. Interestingly, N enhanced the activation of NF-κB and FcRn in a dose-dependent manner (Figures 4D,E), following Western blot analysis with antibodies against FcRn, IκBα, phospho-p65, total-p65. The results showed that the expression of N had no significant effects on the total amount of p65; however, the level of phosphorylated p65 (p-p65) and nuclear p65 increased markedly, and the level of IκBα decreased significantly (Figure 4F). As shown in Figure 4G, pre-treatment of cells with siRIG-I impaired FcRn expression by TGEV N protein in comparison with IPEC-J2 cells transfected with NC siRNA. This result showed that RIG-I may be responsible for TGEV N-protein-induced FcRn expression. In this study, we used confocal microscopy to identify that the TGEV N protein has an effect on the nuclear translocation of the NF-κB subunit p65. We observed the control vector-transfected IPEC-J2 cells and p65-EGFP was mainly present in the cytoplasm. Interestingly, the overexpression of TGEV N could lead to p65-EGFP expression in the nucleus in transfected IPEC-J2 cells (Figure 4G). Taken together, these results demonstrated that TGEV N protein can both significantly activate NF-κB and up-regulate the expression of FcRn.

### Mutations in N Protein Can Reduce FcRn Expression

**FIGURE 5:**
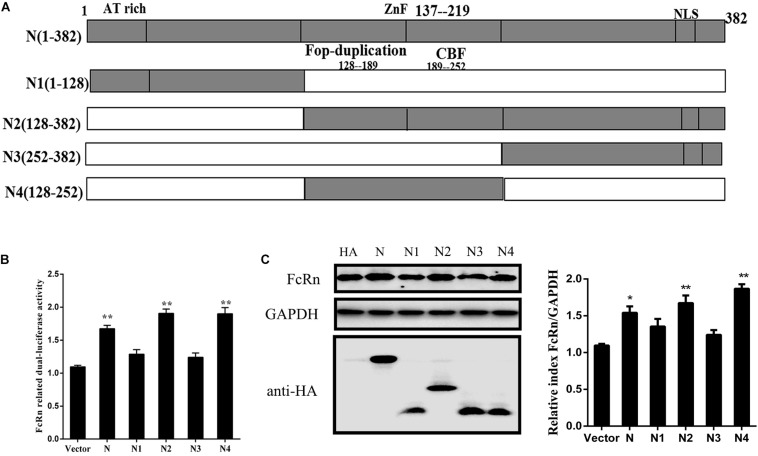
Schematic representation of the TGEV N-protein serial-deletion mutant constructs. **(A)** Schematic representation of the TGEV N-protein serial-deletion mutant constructs. **(B)** IPEC-J2 cells were co-transfected with pRL-TK, FcRn-Luc, and TGEV N deletion-mutant expression plasmids. The cell lysates were harvested for dual luciferase assays at 24 hpi. **(C)** IPEC-J2 cells were co-transfected with TGEV N deletion-mutant expression plasmids for 36 h and protein expression in cell lysates was measured by Western blot using anti-HA antibody and anti-FcRn antibody. The right panel represents the quantification of the bands by densitometry, corrected by the amount of GAPDH. ^∗^*p* < 0.05, ^∗∗^*p* < 0.01.

In order to further explore N functional domains, N protein truncations were expressed in this study. According to the Lasergene DNASTAR Protean 9.0 software prediction, the N-protein contains two nuclear export signal (NES) sequences at aa 221–236 (NES-1), aa 330–353 (NES-2), an RNA-binding region at aa 33–159, a specific SR-rich region at aa 156–198 and a ZnF region at aa 137–219 (Figure 5A). TGEV N protein is mainly located at aa 128–252 and this region has high hydrophilicity and flexibility. In order to study the TGEV N protein, the structure of which is essential for FcRn activation, four truncation mutants were cloned into the eukaryotic expression plasmid, pCAGGS-HA, named as N1(1–128), N2(128–382), N3(252–382), and N4(128–252). The relationship between these mutants and FcRn activation was analyzed through luciferase reporter assays and Western blot. As shown in Figure 5, N4, which contains NES1 and the immune dominant area, compared with the total length of the TGEV N protein, increased the FcRn activation and expression (Figures 5B,C). The result showed that the central region (128–252) of N protein is critical for FcRn activation.

### Recombinant TGEV N Protein Mediated Induced FcRn-Mediated IgG in Polarized IPEC-J2 Cells

**FIGURE 6:**
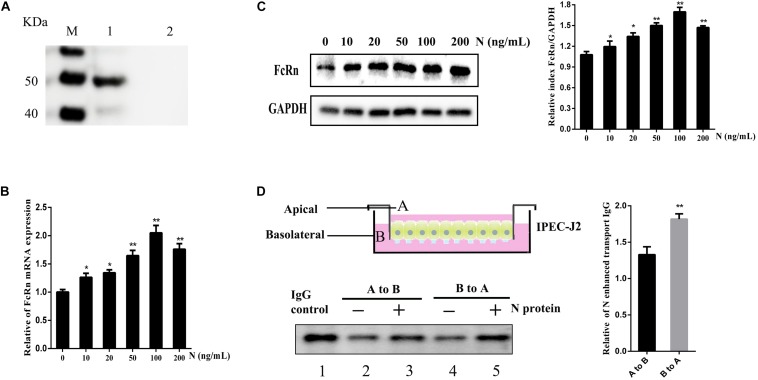
Effects of recombinant TGEV N stimulation on the IgG transcytosis in IPEC-J2 cells. **(A)** Analysis of recombinant TGEV N protein by Western blot. M: Protein marker. 1: TGEV recombinant N. 2: Negative Control. IPEC-J2 cells were treated with recombinant TGEV N protein (0, 10, 20, 50, 100 or 200 ng/mL) for 12 h. **(B,C)** FcRn expression levers were detected by RT-qPCR and Western blot. The right panel represents the quantification of the bands by densitometry, corrected by the amount of GAPDH. **(D)** Transwell system used throughout this study. A, apical; B, basolateral. IPEC-J2 cells were grown in a 12-well transwell plate about 6–7 day until TEER > 1000 Ω/cm^2^. IPEC-J2 cells were stimulated with or without recombinant TGEV N (100 ng/ml) for 12 h. Cells were loaded with porcine biotin-IgG at 37°C in either the apical (lanes 2 and 3) or basolateral (lanes 4 and 5) chamber. Lane 1 represents an IgG. ^∗^*p* < 0.05, ^∗∗^*p* < 0.01.

Transmissible gastroenteritis virus N protein was expressed by Bac-to-Bac baculovirus expression systems, the secreted 49 KDa was verified as we expected by Western blot (Figure 6A). IPEC-J2 cells were stimulated with different doses of recombinant TGEV N protein. We further verified the FcRn up-regulation up to two times by recombinant TGEV N stimulation, as assessed by RT-qPCR and Western blot (Figures 6B,C).

The FcRn protein has been well known to transport IgG bidirectionally in polarized epithelial cells. Therefore, we hypothesized TGEV N protein could alter the role of IgG transcytosis epithelial cells and constructed the transwell system to mimic the porcine intestinal microenvironment using IPEC-J2 cells (Figure 6D). The polarized monolayers (TEER > 1000 Ω/cm^2^) were stimulated with recombinant TGEV N protein (100 ng/mL) for 12 h. Then, porcine biotin-IgG was added to the apical or basolateral surface of a IPEC-J2 cell monolayer, after 3 h at 37°C we assessed the IgG transported to the opposite basolateral or apical chamber, respectively. The IgG H chain was detected in medium by Western blot (Figure 6D, lane 1). Importantly, IgG transport was enhanced 1.3-fold in the apical to basolateral (Figure 6D, lane 3) direction or 1.8-fold in the basolateral to apical (Figure 6D, lane 5) direction by TGEV N in comparison to the mock-treated monolayer (Figure 6D, lanes 2 and 4).

## Discussion

The secretory IgA and IgG play major roles in mucosal anti-infection immunity and are obtained primarily through the polymeric immunoglobulin receptor (plgR) and FcRn-mediated transport. Reovirus, dsRNA (a ligand for TLR3), LPS (a ligand for TLR4), IFN-γ, IL-4, IL-1β, TNF-α, and TGF-β all regulate pIgR expression (Johansen and Brandtzaeg, 2004; Pal et al., 2005; Schneeman et al., 2005). Therefore, LPS, IL-1β, and TNF-α could up-regulate the expression of FcRn via the NF-κB pathway. NF-κB appears to be a positive regulator of FcRn expression. In our previous study, we identified that TGEV infection up-regulated FcRn via the activation of the NF-κB pathway (Guo et al., 2016b). Consistent with this, the replication of the virus is directly related to FcRn expression.

Transmissible gastroenteritis virus infection up-regulates the expression levels of IL-1β, IL-6, IL-8, TNF-α, and TGF-β, which are important factors in chronic inflammation. The expression of FcRn was up-regulated by the inflammatory cytokine TNF-α and IL-1β in a dose-dependent manner, as determined by RT-qPCR (Figures 3A,B), and this was consistent with that observed in a previous study (Liu et al., 2007). MAPK is a highly conserved serine protein kinase in the cytoplasm that mediates a signaling pathway that plays an important role in the body’s inflammatory response. The MAPK family is mainly divided into three classes: extracellular signal-regulated kinases (ERKs), JNKs, and p38 MAPK kinases. Multiple stimulating factors activate the MAPK pathway, including inflammatory factors, growth factors, and cellular responses (Huang et al., 2004). TGF-β dose-dependently induces pIgR production via the p38/MAPK pathway in human bronchial epithelial cells (Ratajczak et al., 2010). Moreover, TGF-β was said to inhibit pIgR production in bronchial epithelium (Gohy et al., 2014). In this study, we observed that TGF-β up-regulates FcRn via the JNK MAPK pathway, perhaps because TGEV activates the JNK MAPK pathway in porcine cells.

The ability of TGEV proteins to stimulate the FcRn promoter was identified by constructing a series of recombinant expression plasmids. However, efficient expression was obtained only from plasmids encoding the N, E, 3a, nsp1, nsp2, nsp5, nsp7, nsp8, nsp9, nsp10, nsp13, and nsp14. The expression of viral protein was not high or related to cell type and state. Among these proteins, N was the most potent NF-κB activator, and it induced FcRn expression. The proteins 3a, nsp1, and nsp5 also enhance but have little influence on NF-κB promoter activity. While nsp2 and nsp14 are also potential NF-κB activators, consistent with that reported in previous studies (Zhou et al., 2017; Wang et al., 2018). Since the essential and multifunctional N protein is fundamental for immune regulation and pathogenesis, we examined it more closely. Porcine epidemic diarrhea virus (PEDV) N protein mediates NF-κB activation through TLR pathways and has functions in cell growth, cell cycle and ER stress (Xu et al., 2013). In fact, the interaction of TGEV N protein with several host cell proteins has been shown (Zhang et al., 2014, 2015). In addition, overexpression of TGEV N protein induces cell cycle arrest (Ding et al., 2014a). Using domain-specific mutations, we determined that the domain at aa 128–252 of the N protein (immunodominant central region) is critical for FcRn activation; it contains a ZnF structure and a SR-rich region. Both the SR-rich truncated proteins of the N protein of SARS coronavirus and PEDV can effectively promote the activation of the NF-κB signaling pathway (Zhang et al., 2007; Cao et al., 2015a).

Epithelial TLR expression plays an important role in the innate and adaptive immune responses. NF-κB is a branch of the downstream signaling pathways of TLR and RLR, which also play important roles in various immune responses. Diverse coronaviruses can escape host immune responses via NF-κB activation through different pathways. PEDV activates NF-κB signaling through TLRs, while mouse hepatitis virus (MHV) does so through RLRs (Li et al., 2010; Cao et al., 2015a). IPEC-J2 cells express mRNA encoding TLR1, TLR2, TLR3, TLR4, TLR6, TLR8, TLR9, TLR10, IL-1α, IL-6, IL-7, IL-8, IL-18, TNF-α, and GM-CSF (Bowie and Haga, 2005; Burkey et al., 2009; Mariani et al., 2009). Rotavirus infection triggers the production of IL-8, IL-6, and TNF-α in IECs by activating the TLR3 and RIG-I pathways (Broquet et al., 2011). Compared with the NC siRNA, TLR3-, TLR9-, RIG-, MyD88-, and TRIF-specific siRNAs inhibited TGEV-induced FcRn activation, while TLR2-, TLR4-, and TLR8-specific siRNAs did not. We speculate that TGEV activates NF-κB via the TLR3, TLR9, and RIG-I pathways. Our results suggest that TLR3, TLR9, and RIG-I may be used to activate the FcRn signaling pathway when TGEV infects IPEC-J2 cells. Recently, our research has shown that PEDV down-regulate the NF-κB signaling pathway to inhibit FcRn by TLR3 and RIG-I pathway in the early stage of infected piglets (Qian et al., 2019). PEDV and PDCoV N protein inhibited IFN-β by RIG-I pathway (Ding et al., 2014b; Likai et al., 2019), and we found that TGEV N protein up-regulated FcRn by RIG-I pathway. Moreover, TGEV infection induced IFN-β production and PEDV inhibited IFN-β production (An et al., 2014; Cao et al., 2015b). These results may be important causes of PEDV being more pathogenic than TGEV.

Transgenic mice that overexpress hFcRn can improve the antigen presentation ability of dendritic cells and enhance the humoral immune response of the body (Végh et al., 2012). FcRn not only transports IgG, but also transports antigen-antibody complexes, which are taken up by antigen-presenting cells (APCs) (Rath et al., 2013). When the pathogen stimulates the mucosal epithelium, the expression of FcRn may be regulated. Simian/human immunodeficiency virus (SHIV) infection decreases FcRn and pIgR expression, which may also be the mechanism responsible for its pathogenesis in rhesus monkeys (Wang and Yang, 2016). On the other hand, TGEV up-regulates FcRn expression, which is potentially what provides the mucosal protection. To observe and measure IgG transcytosis *in vitro*, we applied the transwell system to mimic the porcine intestinal microenvironment, using IPEC-J2 cells to construct the transwell model, our previous research showed that FcRn not only transports specific antibodies across the mucosal epithelium *in vitro*, but also reduces the yield of TGEV (Guo et al., 2016a). We speculated that the up-regulation of FcRn by N protein may mediates more TGEV-specific antibody transcytosis across the mucosal epithelium, thereby enhancing the host’s anti-infective ability. On the other hand, N protein-enhanced FcRn mediates more IgG immune complex transcytosis, followed by antigen uptake by specialized APCs to activate adaptive immune responses. Overall, we elucidated the immune mechanism of TGEV induced FcRn expression and provided a scientific and theoretical basis for the prevention and control of TGEV infection.

## Data Availability Statement

All datasets generated for this study are included in the article/supplementary material.

## Author Contributions

SQ wrote the manuscript. SQ and ZL conceived and initiated the study. SQ, ZG, RC, and KY extracted the dataset. SQ, YC, SL, XM, QH, and ZL performed the analysis. All authors reviewed the manuscript.

## Conflict of Interest

The authors declare that the research was conducted in the absence of any commercial or financial relationships that could be construed as a potential conflict of interest.
